# Dietary Diversity among Chinese Residents during the COVID-19 Outbreak and Its Associated Factors

**DOI:** 10.3390/nu12061699

**Published:** 2020-06-06

**Authors:** Ai Zhao, Zhongyu Li, Yalei Ke, Shanshan Huo, Yidi Ma, Yumei Zhang, Jian Zhang, Zhongxia Ren

**Affiliations:** 1 Wanke School of Public Health, Tsinghua University, Beijing 100091, China; 2 School of Medicine, Tsinghua University, Beijing 100091, China; 3 Department of International Health, Johns Hopkins Bloomberg School of Public Health, Baltimore, MD 21205, USA; zli132@jhmi.edu; 4 School of Public Health, Peking University, Beijing 100191, China; yaleike_bjmu@163.com (Y.K.); shanshanhuo_bjmu@163.com (S.H.); yidima_bjmu@163.com (Y.M.); yumeizhang_bjmu@163.com (Y.Z.); Jianzhang_bjmu@163.com (J.Z.); zhongxiaren_bjmu@163.com (Z.R.)

**Keywords:** COVID-19, household dietary diversity score, dietary behaviors

## Abstract

COVID-19, a Public Health Emergency of International Concern, has imposed enormous challenges on the health system, economy, and food supply and has substantially modified people’s lifestyles. This study aimed to (1) explore the dietary diversity during the lockdown time in China and (2) examine factors associated with dietary diversity including socio-economic characteristics, sources for food and food purchases, and specific dietary behaviors responding to COVID-19 and isolation. A cross-sectional questionnaire-based survey was conducted online in March 2020. Multi-stage sampling was used to recruit participants living in Hubei Province and other parts of China. Dietary diversity was assessed using the Household Dietary Diversity Score (HDDS) and clustering analysis was used to categorize people with different propensities of methods for purchasing or obtaining foods. Logistic regression was used to model the associations among HDDS, participants’ characteristics, approaches to purchase or obtain food, and behaviors adopted to cope with COVID-19. Results: A total of 1938 participants were included in the analysis. The overall mean HDDS was 9.7 ± 2.1, and the median (25th, 75th) was 10 (8, 12). There were relatively low consumptions of fish, legumes, and miscellaneous foods (e.g., processed food like snacks and beverages). After adjusting for age, family income, and geographic regions, people living in places where laboratory confirmed COVID-19 cases were above 500 (OR_adjusted_ = 0.79, 95%CI 0.65, 0.96), or living in Hubei Province (OR_adjusted_ = 0.60, 95%CI 0.39, 0.93) had a lower HDDS. During isolation time, the most common sources for food and food purchases were in-house storage and in person grocery shopping. More than half of the participants (55.9%) purchased food at least once via online ordering and delivery services. There was no significant difference in HDDS among people with distinct dependences on different ways to obtain or purchase food (i.e., dependence on in-person grocery shopping, dependence on both in-house storage and in-person grocery shopping, or dependence on online food purchasing). We also identified a total of 37.7% participants who consumed certain foods or nutritional supplements to cope with COVID-19, which included vitamin C, probiotics, other dietary supplements, alcohol, and vinegar. People who reported these specific dietary behaviors had a significantly higher HDDS (OR_adjusted_ = 1.23, 95%CI 1.02, 1.45) than those who did not do so. This study revealed an overall good dietary diversity among the studied Chinese residents during the COVID-19 pandemic. However, we observed a lower dietary diversity among people living in areas with a high number of confirmed COVID-19 cases. Online ordering and delivery services were popular and could serve as a feasible method to obtain and purchase food, contributing to ensure diversified diets during the time of lockdown. Certain dietary behaviors associated with COVID-19 were also identified and had significant impacts on HDDS.

## 1. Introduction

The Coronavirus Disease 2019 (“COVID-19”) has emerged rapidly as a respiratory disease caused by the severe acute respiratory syndrome coronavirus 2 (SARS-CoV-2), responsible for an outbreak that took place in December 2019 in Wuhan, China [1]. Due to the highly contagious nature of this novel coronavirus, Hubei and other provinces in China have adopted several unprecedented measures to control the transmission of COVID-19 including the suspension of public transportation, restricted access to communities, closure of public spaces, and management such as hospitalization and isolation of confirmed and suspected cases [2]. Chinese residents living both in and outside Hubei had been required to stay at home to self-isolate since 27 January 2020. By 15 April 2020, this virus had reached a total of 213 countries, resulting in more than 1.9 million laboratory-confirmed infections and 120,000 deaths [3]. Consequently, more than 40 countries and areas including Italy and regions in the United States have started implementing similar lockdown and “shelter in place” measures as those in China since 1 April. Even though most supermarkets and grocery stores remain open during the isolation period, food supply as well as safe and adequate access to foods have become major concerns regarding the essential needs among the general populations due to the restrictions of transportation, self-reduction of outside activities, and lack of enough labor force, especially in areas severely affected by COVID-19. On 31 March, the United Nations stated that COVID-19 has threatened the food supply chain globally, and it is estimated that this situation may worsen in April and May [4].

Foods contain essential nutrients and important phytochemicals that not only support basic biological functions in living organisms, but also exert protective and complementary effects on preventing and treating diseases including infections [5,6,7]. Although no nutrients have been scientifically proven to specifically benefit the prevention or treatment for COVID-19, a balanced and diversified diet is, undoubtedly, crucial in maintaining a properly functional immune system and providing sufficient nutrients for recovery [8,9]. However, as above-mentioned, restriction in social activities and mobility impose potential barriers in people’s access to food. On the other hand, when a stressful situation of this magnitude arises, people often experience substantial changes in their emotions, which may subsequently lead to modifications or development of some dietary behaviors such as seeking relief or cure from certain foods [10]. Those alterations in activities and behaviors during a pandemic period collectively influence and potentially impair food diversity. Currently, little is known about the dietary diversity during COVID-19 pandemic.

China has been experiencing a rapid growth of online food ordering and delivery services in recent years. According to “Chinese Restaurant Super Digital Time Insight 2020”, in 2019, the total value of food delivery service reached 727.4 billion Chinese yuan [11]. Especially, in the recent self-isolation and “stay at home” time, online food shopping has gained in popularity because of its apparent role in reducing unnecessary person to person contact. In China, delivery services including food delivery have resumed since 28 January, and by early February, most delivery services have gone back to normal and cover most parts of China [12]. People are able to purchase not only processed foods like fast foods and restaurant takeout, but also fresh produce (i.e., fruits and vegetables), fresh meat, and grains via online food ordering and delivery services, thereby possibly increasing food accessibility. For this reason, proper measurements and evaluations are needed to investigate the potential benefits of this relatively novel and modern approach for food purchasing in maintaining dietary diversity during isolation time.

To the best of our knowledge, no study has reported on dietary diversity during isolation time in China and other areas. This study was conducted via a quick online survey to assess dietary diversity among Chinese residents during the time of isolation and “staying at home” due to COVID-19 and to explore its associated factors.

## 2. Materials and Methods

### 2.1. Study Design and Participants

**Figure 1:**
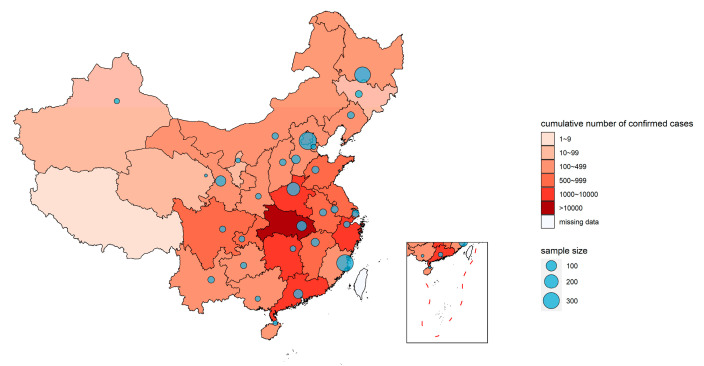
Geographical distribution of participants in the study. The color of the map indicates the cumulative number of confirmed cases in each province by the end of 31 March, according to the report from the Chinese Disease and Control Center [13]. Bubble size in the bubble plot represents the sample size of every investigation point.

This cross-sectional study was composed of a questionnaire-based survey conducted in March via Chinese e-questionnaires using Wenjuan xing (Wenjuan xing Tech Co. Ltd., Changsha, China), a widely used online platform in China and distributed via the most common social media used in China “Wechat” (Tencent Inc. Shenzhen, China). A multistage sampling method was used. We purposefully selected and included people living in Hubei Province, the hard-hit area by COVID-19 outbreak in early 2020, China and people living in north, south, and central China. Then, the “snowball sampling” method was used to recruit more participants. To identify the respondents’ attitude in completing the questionnaire, one question “Did you seriously look through the questions and answer the questions according to your actual situation” appeared in the middle part of the questionnaire. A total of 2021 Chinese residents participated in this survey. The inclusion criteria were (1) living in Mainland China, and (2) aged from 18–80 years old. The exclusive criteria were (1) people infected with COVID-19, (2) who have disease which impacts of normal eating, and (3) who did not seriously respond to the questions in this survey. After all the data had been collected, the final analysis included 1938 participants (people who did not seriously respond to the questions (*n* = 10), who were living outside Mainland China (*n* = 12), who were aged <18 y or >80 y (*n* = 55), who were infected with COVID-19 (*n* = 2), or who had missing data on key questions (*n* = 3) were excluded. One participant self-reported no food intake in the past several days was also excluded). The geographical distribution of participants is displayed in Figure 1.

### 2.2. Data Collection

The questionnaire contained four parts: socio-demographic characteristics, household food diversity, sources of the food (methods for purchasing or obtaining food), and specific dietary behaviors to cope with COVID-19.

The number of confirmed cases by 31 March in each province in China was obtained from the Distribution of COVID-19 Report that is accessible on the Chinese Center for Disease Control and Prevention website (imported cases not included) [13]. The isolation status (still working outside home, self-isolation at home, close contact isolation, and infected with COVID-19) and frequencies of outside activities were also recorded.

We evaluated dietary diversity using Household Dietary Diversity Score (HDDS), a measure that reflects household food accessibility [8]. In total, the intakes of 12 food groups over the last 24 h (food eaten outside and at home) were investigated including (1) cereals; (2) roots and tubers; (3) vegetables; (4) fruits; (5) meat, poultry, and offal; (6) eggs; (7) fish and seafood; (8) pulses, legumes, and nuts; (9) dairy products; (10) oils and fats; (11) sugar and honey; and (12) miscellaneous such as condiments, snacks, and beverages. Values for each food group were assigned as “0” or “1”, “0” for not-consumed and “1” for consumed in the last 24 h. Proportions of participants who consumed each food group were calculated. Also, the total scores of 12 groups were calculated for estimating the dietary diversity, which could range from 0 to 12. A higher score indicates a higher dietary diversity.

To investigate food sources during isolation time, we asked participants to select from four of the most common approaches to obtain or purchase different kinds of foods (12 food items in the HDDS) based on the ones that they had used. The four approaches were (1) using food stored in house before self-isolation; (2) purchasing food from traditional markets and grocery stores in person; (3) using online food ordering and delivery services (including purchasing both raw ingredients and prepared meals from restaurants); and (4) dependent on government-or community-based food distribution.

We also explored certain dietary strategies that participants adopted to cope with COVID-19 by using several “yes” or “no” questions such as “whether participants increased consumption of vitamin C, probiotics, any other kinds of dietary supplements, Chinese herbs, vinegar, and alcoholic beverages or not”. In addition, one open question was used to identify any other previously unspecified foods consumed to cope with COVID-19.

### 2.3. Ethics

Participants answered the questionnaire anonymously. Informed consent was obtained from participants who confirmed their willingness to participate voluntarily prior to the survey.

### 2.4. Statistics

Data were analyzed using the software SAS version 9.4 (SAS Institute, Cary, NC). HDDS were presented as means ± standard deviation (SD) and median (25th, 75th percentiles) and were tested using the independent T-test and Variance analysis. The proportions of participants who consumed each food group in the last 24 h were presented as numbers (percentage). To explore the participants’ behaviors in acquiring foods, we first calculated the propensity of choosing different approaches for obtaining or purchasing foods among our participants. For each food group, there were four pre-defined approaches (in-house storage, in-person grocery shopping, online shopping, or government and community-based food distribution programs) to obtain the food. We gave one point when participants purchased or obtained food from each food group with one of the four approaches. The total propensity score of utilizing each approach to obtain or purchase 12 groups of food for individual participants could range from 0 to 12. Based on the total propensity scores of each approach, major patterns of purchasing and obtaining foods were identified with the Hierarchical Cluster and K-means clustering method. We first randomly selected 30% of participants that were then used in the hierarchical cluster analysis to determine the proper number of clusters. Three clusters were identified by K-means cluster analysis. People in cluster 1 showed dependence on in-person grocery; people in cluster 2 depended on both in-person grocery and in-house storage; and people in cluster 3 showed more dependence on online food ordering and delivery services.

HDDS was divided into two groups based on its median (high (>=median) and low (<median) HDDS). Logistic regression was used to model the associations among HDDS, participants’ characteristics, approaches for food purchasing/sourcing, and certain dietary strategies to cope with COVID-19. We adjusted for potential socio-demographic confounders (age, household average annual income, and geographic region) in the fully adjusted models. A *p*-value < 0.05 was considered statistically significant in all analyses.

### 2.5. Heat Map

The heat map of the geographical distribution of COVID-19 cases in Mainland China and the bubble plot of study sample sizes were created using R version 3.6.3 with the packages “ggplot2”, “maptools”, “rgdal”, and “sp” [14]. The base map was obtained as a shape file from the National Fundamental Geographic Information System, China. Cumulative numbers of confirmed cases of the COVID-19 were calculated at the province (autonomous regions or municipalities directly under the central government included) level and coded with color. The lighter the color, the fewer the cases. Bubble sizes in the bubble plot proportionally represent the numbers of participants at each sampling point.

## 3. Results

### 3.1. Household Dietary Diversity Score (HDDS) Status of Participants

A total of 1938 participants were included in the analysis. The overall average HDDS was 9.7 ± 2.1, median (25th to 75th) is 10 (8 to 12). Proportions of participants who consumed each food group in the last 24 h are shown in Table 1. The cereals group had the highest consumption, whereas the fish, legumes, and miscellaneous had relative low consumptions.

### 3.2. HDDS among Participants with Different Socio-Demographic Characteristics

Compared to people aged above 45, those aged from 18–45 years have a diet with a lower diversity score. Living in urban areas was associated with a significantly higher HDDS than those living in rural areas. Meanwhile, the HDDS increased with family income (Table 2).

### 3.3. Sources for Obtaining and Purchasing Foods during Isolation Period

**Figure 2:**
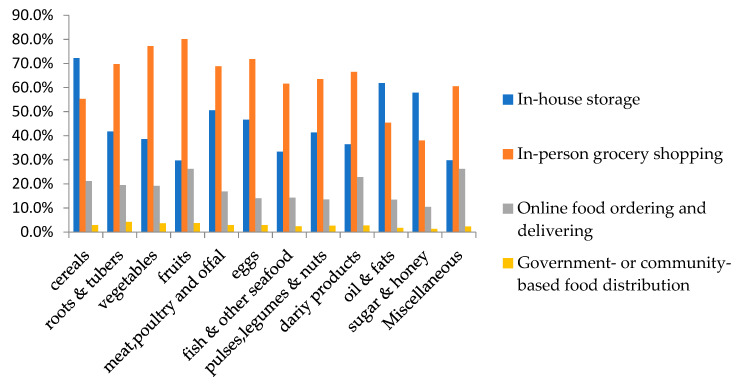
Participants’ choices of approaches to obtain or purchase foods during the isolation period (%).

During the isolation time, the most common food sources were in-house storage and in-person grocery shopping. The participants’ choices of approaches to obtain or purchase foods during isolation are shown in Figure 2. The most frequently purchased foods for in-person grocery shopping were fruits (80.1%), vegetables (77.2%), and eggs (71.8%). A total of 55.9% people used online ordering and delivery services at least once. Miscellaneous food (such as condiments, fast food, and snacks) (26.2%), fruits (26.2%), and dairy products (22.8%) were the most commonly purchased foods via online food ordering and delivery services, whereas oil and fats and sugar and honey were the least purchased.

### 3.4. Dietary Behaviors Coped with COVID-19

There were 722 (37.7%) participants intentionally consumed dietary supplements, Chinese herbs, or specific foods because of COVID-19. A total of 31.2% of participants consumed various dietary supplements or Chinese herbs (18.2% for vitamin C, 11.7% for probiotics, 8.0% for other dietary supplements, and 9.6% for Chinese herbs) to cope with COVID-19. Meanwhile, there were 10.6% and 16.0% of participants who once purposely consumed alcohol and vinegar to cope with COVID-19.

### 3.5. Factors Associated with HDDS

HDDS was further divided into two groups based on its median value (HDDS = 10) and used as a binary outcome (<10 = 0 = low HDDS; >=10 = 1 = high HDDS). Logistic regression was used to explore the associations among HDDS and the factors listed in Table 3. We found that people who lived in the places where laboratory confirmed COVID-19 cases were above 500 or in the Hubei Province had significant lower odds of high HDDS. Isolation status, frequencies of outdoor activities, and frequencies of going out to purchase food were not associated with HDDS.

Based on K-means clustering analysis, participants were clustered into three groups. People in cluster 1 showed dependence on in-person grocery shopping for food; people in cluster 2 depended on both in-person grocery and in-house storage; and people in cluster 3 depended mostly on online food shopping. HDDS was not associated with dependences on different approaches to purchase food. Interestingly, people who reported adoptions of certain dietary behaviors to cope with COVID-19 had higher odds of being in the high HDDS group than people who did not report doing so.

## 4. Discussion

As a declared Public Health Emergency of International Concern, COVID-19 has rapidly spread from Wuhan, Hubei to other parts of China and countries worldwide. This pandemic imposes enormous challenges on the health system, economy, and food supply globally and locally [4,15]. Meanwhile, COVID-19 and subsequent measures to prevent its spread have substantially changed people’s lifestyle. There have been several studies reporting on people’s behaviors during pandemic such as consciously avoiding crowded places and wearing masks [16]. On the other hand, psychologically, people may feel anxious during outbreaks of infectious diseases, which impacts their sleep quality and other health related quality of life [17]. Nonetheless, there are not many, if any, studies that have examined dietary diversity during an epidemic or a pandemic. We believe that the current study is the first to report dietary diversity among Chinese residents during the COVID-19 pandemic period. Our study showed an overall good dietary diversity in the study sample, though there was a reduction in diversity in places where more COVID-19 cases were confirmed. To our best knowledge, this is also the first study to explore potential factors associated with dietary diversity in a pandemic. We found dietary diversity did not vary across different approaches to obtain or purchase foods, which provides evidence supporting that online food ordering and delivery services could achieve a similar dietary diversity as in-person groceries and in-house storage do. In addition, several specific dietary behaviors were identified during the COVID-19 outbreak and they contribute to higher odds of high dietary diversity.

### 4.1. Dietary Diversity in Participants

To ensure a good nutritional status during lockdown, as early as 8 February, the National Health Commission of China published the “Dietary Guidelines for the Prevention and Treatment of Novel Coronavirus”, which advocates a diverse diet for the general public [18]. The World Health Organization (WHO) recommends that “If you must stay at home, maintain a healthy lifestyle-including proper diet, sleep, exercise and social contacts” [19]. However, the degree to which people have followed these recommendations is unknown. In this study, we focused on dietary diversity and found an overall relatively high diet diversity (average HDDS = 9.7 ± 2.1, 25th to 75th were 8 to 12) when the “shelter in place” was in effect in China (the current study was carried out in March). This number is higher than the HDDS reported pre-COVID-19 in Indonesia (9.1), South Africa (8.0), and rural Cambodia (4.7) [20,21,22]. The HDDS is usually used to reflect food accessibility [8]. Access to food is a critical component of food security and plays a vital role in health and health disparities [23]. Several studies have demonstrated that the HDDS is inversely associated with the risk of malnutrition. For instance, one study in South Africa showed that children with high HDDS (>8) had a significantly lower wasting rate [21]. In Ethiopia, when the HDDS drops below four, adolescents are likely to suffer from underweight [24]. It is encouraging that the HDDS reported in the current study suggested an overall good food accessibility and low risk of malnutrition among the Chinese residents surveyed during the COVID-19 pandemic.

Among the 12 food groups assessed in the HDDS, fish, legumes, and miscellaneous foods had relatively low consumption during the last 24 h. Miscellaneous foods include most seasoning, snacks, beverage, and instant meals that can be highly processed. They tend to be deprived of essential nutrients, are energy dense, and are less healthy compared to freshly home-made meals [25]. Many studies have shown that the high consumption of such foods contributes to excessive weight gain and increased risks of chronic conditions such as cardiovascular diseases and obesity [26,27]. These pre-existing conditions have been reported to increase the severity of COVID-19 infection and potentially worsen the disease outcome [28,29]. Noteworthy, in our study, the consumption of miscellaneous food was relatively low (56.9% participants), which may lower the total dietary diversity score. However, as discussed above, low consumption of processed food may also bring potential health benefits. Further investigations are needed to measure the consumption of processed foods and its effects on health during disease outbreaks.

Insufficient intake of fish and legumes in the Chinese population were also observed in a previous study conducted before the COVID-19 pandemic [30,31]. However, due to cultural preferences in the Chinese population for fresh foods that are likely to be negatively affected by the lockdown policy, it is unsurprising that the intake of food like fish and seafood, whose tastes and flavor largely depend on the freshness of the raw materials, may decrease [32]. To improve the residents’ access to fresh fish, Wuhan city implemented a working program to promote the consumption of fresh fish in every local community on 12 March [33]. Legumes including fresh beans, soybeans, and pulses provide fiber, protein, vitamins, minerals, and phytochemicals such as phytosterols that have been suggested to modulate immune system and exert protective effects against inflammation and oxidative stress [34,35]. In addition, because legumes and fish are both excellent sources of high-quality protein and low in fats, interventions and policies are needed to encourage the inclusion of these two food groups to achieve more balanced and diversified diets rich in nutrients, in particular, during pandemics like COVID-19.

### 4.2. Factors Associated with Dietary Diversity

Participants in this study were distributed across 31 provinces and cities in China. We observed a slightly lower HDDS among people living in the areas where more COVID-19 cases had been confirmed. Similar concerns were raised in the areas hit hardest by Ebola in 2014 and 2015. In Guinea and Liberia, where food became unavailable and food insecurity rose rapidly, people shifted toward a less nutritious diet with limited diversity [36,37]. Those findings indicate that areas with high incidence of infectious diseases like COVID-19 should pay more attention to dietary diversity, especially in the areas where people are required to self-isolate at home and food supply mostly depends on government or community-based food distribution programs. Meanwhile, more novel strategies should be developed to meet the needs of adequate and diverse food supply in this particular time.

As reported by the study participants, in-house storage and in-person grocery shopping were still the major sources of food supply during lockdown. Meanwhile, online food ordering and delivery services have gradually become an important method to purchase food in the daily lives of Chinese residents [11]. In this study, 55.9% participants used online ordering and delivery services at least once. The clustering analysis grouped study participants into three clusters based on their methods for purchasing or obtaining foods. When comparing the HDDS among people with different preferences for purchasing foods, we found that participants who purchased food primarily via online food shopping, or who depended on in-house storage and in-person grocery shopping, or purchased the most food in person from grocery stores had similar HDDS. In addition, we observed that fruits and dairy products were the most common food purchased via online services, which are also the food groups recommended in the Chinese Dietary Recommendation responding to COVID-19 [18]. Therefore, online ordering and delivery services may serve as a feasible solution to sustain stable food supply and adequate food access in the COVID-19 pandemic as it can maintain dietary diversity and potentially reduce the spread of virus by limiting person to person contact. On 10 February, the Chinese State Administration for Market Regulation promulgated specific regulations corresponding to the online food ordering and delivery services, which requires checking the deliveryman’s temperature daily, disinfecting the equipment, requesting staff wear gloves and masks during delivery services, etc. [38]. These requirements may be helpful in curtailing the COVID-19 pandemic, but further evaluations on their effectiveness in slowing the growth of COVID-19 cases are still needed. Furthermore, other potential concerns on food delivery such as the risk of food contamination should also be taken into consideration.

Disease outbreaks often influentially impact health-related behaviors. Joseph et al. reported that comparing health-seeking behaviors in the post-and pre-SARS epidemic period in 2003, people were more likely to adopt a healthier diet, especially among those who were worried about contracting the virus [39]. Interestingly, our study found that 31.2% of our participants intentionally consumed vitamin C, probiotics, and other dietary supplements to cope with the novel coronavirus outbreak. Meanwhile there were 10.6% and 16.0% of participants once purposely drinking alcohol and vinegar, respectively. These behaviors are likely to be caused by rising concerns in this stressful time of COVID-19 pandemic. Intriguingly, we observed higher HDDS among participants with those behaviors. We infer that it may be because the people who have these behaviors may also pay more attention to diet. However, it should be noted that none of these behaviors has been officially recommended and they are not supported by rigorously tested scientific evidences. For instance, the idea of drinking high liquor to prevent viral infection emerged soon after several Chinese scientists announced that 75% medical alcohol could inactivate the virus. Although the Chinese government had already dismissed this rumor since 22 January [40], there were still more than 10% of the studied population purposely drinking more alcohol. This behavior has also been reported in many other countries. According to the report from Tasnim News Agency on 27 March, there have been at least 2197 people poisoned and 244 deaths in Iran due to the consumption of toxic alcohol (methanol based beverage) that was believed to prevent COVID-19 by some people [41]. Another interesting phenomenon is that 16% of the studied participants had drunk more vinegar to fight against the virus, which could also be seen in 2003 during the SARS pandemic [42]. Unfortunately, no scientific evidence has proven the effectiveness of drinking vinegar in lowering the risks of viral infection or mortality.

### 4.3. Limitation

Based on the online survey methodology, HDDS was used in this study for its convenience, and to some extent, it could reflect the food accessibility and predict the risk of malnutrition. However, it does not quantify the amount of actual food intake and, therefore, we could not estimate the level of nutrient sufficiency or deficiency. Additionally, according to a validation study, the current components of indicators in HDDS do not provide a reliable way to reflect the household-level access to food [43]. No previous Chinese study has used this indicator, so the reliability was unknown in the Chinese population. In addition, the HDDS was designed with an interview methodology and there is no pre-test of its efficiency on self-completion. The e-based questionnaire may also lead to selection bias. The majority of participants in the current survey were young and highly educated. Elders and people with relatively low socio-economic status could not be easily reached in this study, however, they are often the more vulnerable groups during the COVID-19 pandemic. Further studies should focus on these populations and provide specific strategies to ensure their nutritional status. Another unfortunate limitation in current study is that we could not obtain the anthropometric data, hence the direct health outcomes could not be observed. The impacts of dietary intake during pandemic on health such as weight change and immune functions need to be evaluated.

## 5. Conclusions

This study reported on dietary diversity in Mainland China during the COVID-19 pandemic and revealed a generally good dietary diversity among the Chinese residents studied. However, people living in areas with a high number of confirmed COVID-19 cases had a lower HDDS. Several dietary behaviors used to cope with COVID-19 were identified including increased consumption of vitamin C, probiotics, other dietary supplements, alcohol, and vinegar. People with these behaviors had a higher HDDS. During lockdown, in-house storage and in-person grocery shopping were the primary ways to obtain food. There was no difference in the HDDS among people who depended more on in-person grocery shopping, in-house storage, or online ordering and delivery services to obtain or purchase foods.

Based on the current study, we have proposed the following recommendations. (1) Evidence based dietary recommendations and health education are needed to encourage a more balanced and diversified diet and to prevent inappropriate eating behaviors, especially in areas severely impacted by COVID-19 and the lockdown policy. (2) Cautiously monitored and regulated online ordering and delivery services could serve as a feasible method for food purchases, which ensures dietary diversity. (3) More studies are urgently needed to identify potential nutritional concerns for different populations with various health conditions and socio-demographic characteristics during pandemics and provide corresponding strategies.

## Figures and Tables

**Table 1:** Food groups consumption in the last 24 h (*n*, %).

Food Groups	Consumed	Not-Consumed
Cereals	1896 (97.8)	42 (2.2)
Tubers and roots	1549 (79.9)	389 (20.1)
Vegetables	1913 (98.7)	225 (1.3)
Fruits	1823 (94.1)	115 (5.9)
Meat, poultry and offal	1547 (79.8)	391 (20.2)
Fish and other seafood	1110 (57.2)	828 (42.7)
Eggs	1801 (92.9)	137 (7.1)
Pulses, legumes, and nuts	1360 (70.1)	578 (29.8)
Dairy products	1545 (79.7)	393 (20.3)
Oils and fats	1817 (93.7)	121 (6.2)
Sugar and honey	1345 (69.4)	593 (30.6)
Miscellaneous ^a^	1103 (56.9)	835 (43.1)

^a^ Miscellaneous foods include condiments and most processed foods like snacks and beverages.

**Table 2:** Household Dietary Diversity Score (HDDS) among participants with different socio-demographic characteristics.

		*n* (%)	HDDS (Mean ± SD) ^b^	*p*
Gender	Male	665 (34.3)	9.68 ± 2.61	0.275
	Female	1273 (65.7)	9.72 ± 2.04	
Age group(y)	18–45	1620 (83.6)	9.65 ± 2.10	<0.001
	>45	318 (16.4)	9.97 ± 1.89	
Education level	Senior high school or under	219 (11.3)	9.60 ± 2.23	0.121
	Bachelor degree	1464 (75.5)	9.76 ± 2.03	
	Master degree or above	255 (13.2)	9.49 ± 2.08	
Family annual income (Chinese yuan)	<30 thousands	206 (10.6)	8.98 ± 2.43	<0.001 ^a^
	30–100 thousands	690 (35.6)	9.69 ± 2.02	
	100–300 thousands	750 (38.7)	9.80 ± 1.97	
	>300 thousands	292 (15.1)	10.02 ± 2.06	
Family size (people living in the same household during isolation)	<3	979 (50.5)	9.73 ± 1.97	0.887
	3–5	760 (39.2)	9.68 ± 2.09	
	>5	197 (10.2)	9.68 ± 2.41	
Geographic Region	Urban	414(21.4)	9.78 ± 2.04	0.003
	Rural	1524(78.6)	9.44 ± 2.15	
Pregnant or lactating women in the household	Yes	44 (2.3)	9.80 ± 2.37	0.770
	No	1894 (97.7)	9.70 ± 2.06	
<5 y children in the household	Yes	325 (16.8)	9.86 ± 2.12	0.143
	No	1613 (83.2)	9.67 ± 2.05	
>65 y elders in the household	Yes	805 (41.5)	9.70 ± 2.06	0.262
	No	1133 (58.5)	9.66 ± 2.03	

^a^*p* for trend. ^b^ HDDS = Household Dietary Diversity Score. y = year

**Table 3:** Odds of high HDDS comparing participants differ in geographic regions, approaches to obtain and purchase foods, frequency of outdoor activity, and dietary behaviors to cope with COVID-19.

		*n* (%)	OR (95% CI)	OR_adjust1_ (95% CI) ^a^
Participants characteristics				
Geographic regions by case number	<500case/province ^b^	987 (50.9)	1	1
	>500case/province ^b^	862 (44.5)	0.84 (0.70, 1.01)	0.79 (0.65, 0.96)
	Hubei	89 (4.6)	0.58 (0.38, 0.90)	0.60 (0.39, 0.93)
Status during isolation	Self-isolation	1254 (64.7)	1	
	Working outside	684 (35.3)	1.04 (0.86, 1.25)	0.95 (0.78, 1.15)
Total of going out times	0–2×/week	401 (20.7)	1	1
	3–4×/week	916 (47.3)	1.13 (0.89, 1.43)	1.06 (0.84, 1.35)
	≥5×/week	621 (32.0)	1.01 (0.78, 1.30)	0.91 (0.70, 1.78)
Frequencies of going out for food purchase	0–2×/week	417 (21.5)	1	1
	3–4×/week	1125 (58.0)	1.20 (0.96, 1.51)	1.15 (0.92, 1.45)
	≥5×/week	396 (20.4)	1.06 (0.80, 1.40)	0.97 (0.74, 1.29)
Food purchasing behaviors ^c^				
	Cluster1	320 (16.5)	1	1
	Cluster2	752 (38.8)	1.03 (0.79, 1.34)	1.03(0.79, 1.35)
	Cluster3	866 (44.7)	1.12 (0.87, 1.45)	1.11(0.86, 1.44)
Dietary behaviors in COVID-19 ^d^				
Reported dietary behaviors to cope with COVID-19	No	126 (62.7)	1	1
	Yes	722 (37.2)	1.27 (1.05, 1.53)	1.23 (1.02, 1.45)

^a^ Adjusting for age, family average annual income, and geographic region (rural or urban). ^b^ All provinces in Mainland China except Hubei. ^c^ Based on k-means clustering analysis, participants were clustered into three clusters. People in cluster 1 mainly depended on in-person grocery shopping to purchase food; people in cluster 2 depended on both in-person grocery shopping and in-house storage; and people in cluster 3 depended mostly on online food ordering and delivery services. ^d^ Certain dietary behaviors include increased intake of vitamin C, probiotics, other dietary supplements, alcohol andvinegar with the intention to cope with COVID-19.
